# The predictive power of geographic health care utilization for unintentional fatal fall rates

**DOI:** 10.1186/s12889-022-12731-x

**Published:** 2022-02-16

**Authors:** Matthew Gordon Crowson, Jason A. Beyea, Justin Cottrell, Faisal Karmali, Giovanni Lampasona, James E. Saunders, Richard F. Lewis

**Affiliations:** 1 Department of Otolaryngology-Head & Neck Surgery, Massachusetts Eye & Ear, 243 Charles Street, Boston, MA 02114 USA; 2 Department of Otolaryngology-Head & Neck Surgery, Harvard Medical School, Boston, MA USA; 3 Department of Surgery, Division of Otolaryngology-Head and Neck Surgery, Kingston Health Sciences Centre, Queen’s University, Kingston, Ontario Canada; 4 ICES Queen’s, Queen’s University, Kingston, Ontario Canada; 5 Department of Otolaryngology-Head & Neck Surgery, University of Toronto, Toronto, Ontario Canada; 6 Faculté de Médecine et des Sciences de la Santé, l’Université de Sherbrooke, Sherbrooke, Quebec Canada; 7 Department of Surgery, Division of Otolaryngology-Head and Neck Surgery, Dartmouth-Hitchcock Medical Center, Hanover, NH USA

## Abstract

**Background:**

Falls are the leading cause of fatal and nonfatal injuries among adults over 65 years old. The increase in fall mortality rates is likely multifactorial. With a lack of key drivers identified to explain rising rates of death from falls, accurate predictive modelling can be challenging, hindering evidence-based health resource and policy efforts. The objective of this work is to examine the predictive power of geographic utilization and longitudinal trends in mortality from unintentional falls amongst different demographic and geographic strata.

**Methods:**

This is a nationwide, retrospective cohort study using the United States Centers for Disease Control (CDC) Web-based Injury Statistics Query and Reporting System (WISQARS) database. The exposure was death from an unintentional fall as determined by the CDC. Outcomes included aggregate and trend crude and age-adjusted death rates. Health care utilization, reimbursement, and cost metrics were also compared.

**Results:**

Over 2001 to 2018, 465,486 total deaths due to unintentional falls were recorded with crude and age-adjusted rates of 8.42 and 7.76 per 100,000 population respectively. Comparing age-adjusted rates, males had a significantly higher age-adjusted death rate (9.89 vs. 6.17; *p* <  0.00001), but both male and female annual age-adjusted mortality rates are expected to rise (Male: + 0.25 rate/year, R^2^= 0.98; Female: + 0.22 rate/year, R^2^= 0.99). There were significant increases in death rates commensurate with increasing age, with the adults aged 85 years or older having the highest aggregate (201.1 per 100,000) and trending death rates (+ 8.75 deaths per 100,000/year, R^2^= 0.99). Machine learning algorithms using health care utilization data were accurate in predicting geographic age-adjusted death rates.

**Conclusions:**

Machine learning models have high accuracy in predicting geographic age-adjusted mortality rates from health care utilization data. In the United States from 2001 through 2018, adults aged 85+ years carried the highest death rate from unintentional falls and this rate is forecasted to accelerate.

**Supplementary Information:**

The online version contains supplementary material available at 10.1186/s12889-022-12731-x.

## Background

Falls are the leading cause of fatal and nonfatal injuries among adults over 65 years old [1]. In a cohort from the United States in 2014, 28.7% of adults aged 65 years or older reported falling at least once in the past year which resulted in approximately 29 million fall events [1]. Of those adults who fell, 37.5% required medical care as a result of the fall [1]. Analogous studies from other countries such as China and the Netherlands have also reported a high risk of fall and subsequent mortality in older adults [2, 3]. Numerous risk factors for falls have been identified and include the presence of gait and balance disorders, medical co-morbidities, neurologic disorders, cognitive function, and environmental hazards [4–12]. The economic burden for direct medical cost of injuries and deaths from falls is substantial with annual costs eclipsing billions of dollars [13, 14].

Given the high prevalence of falls and associated mortality, significant interest in falls prevention, awareness, and public health programming have emerged. Guidelines from the American and British Geriatrics Societies [15], the Stopping Elderly Accidents, Deaths, and Injuries (STEADI) initiative from the Centers for Disease Control (CDC) [16, 17], and various grassroots efforts to promote exercise and home safety interventions [18–20] have been deployed to help curb falls rates. Prior research using administrative data has characterized demographics and falls mortality data over time periods when these public health programs have been implemented, demonstrating a persistent increase in fall mortality rates [21, 22].

The increase in fall mortality rates is likely multifactorial. With a lack of key drivers identified to explain rising rates of death from falls, accurate predictive modelling can be challenging, hindering evidence-based health resource and policy efforts. This study investigates the predictive power of machine learning models trained on geographic healthcare utilization intensity metrics for producing accurate predictions of age-adjusted death rates derived from the Dartmouth Atlas Project. To our knowledge, no studies have combined a longitudinal analysis with predictive analytics to characterize the trends in death rates in demographic and geographic strata. Improved predictive power, and knowledge gained from identifying health care utilization patterns that influence fall mortality rates can inform enhanced falls prevention strategies. Lastly, the economic burden of mortality from unintentional falls is estimated.

## Methods

### Data sources

The CDC Web-based Injury Statistics Query and Reporting System (WISQARS) database was mined for mortality event data attributed to an ‘unintentional fall’ mechanism (available at: https://www.cdc.gov/injury/wisqars/index.html). The CDC compiles mortality data from the National Center for Health Statistics (NCHS), and their own annual mortality data. The NCHS mortality data are based on International Classification of Disease codes (ICD-10). The ICD-10 code corpus includes specific codes for types of disease, medical procedures, and causes of death. The underlying cause of death examined in this study, ‘unintentional falls,’ was isolated from the annual mortality data to determine the numbers of attributable deaths. Other variables mined from WISQARS included binned age, sex, race, and geographic features for the deceased. No limits were placed on age, sex, race, or geographic case parameters.

Age-adjusted rates were utilized in this study to draw comparisons between demographic and geographic groups. The population data is from the U.S. Census Bureau in concert with the NCHS. The population estimates were used by NCHS to calculate mortality rates based on the death data.

The WISQARS database enables the calculation of estimated direct medical and lifetime work-related costs resulting from death events indexed to 2010 national injury and fatality data. The direct medical and lifetime work-related costs are calculated using an economic framework [23, 24]. Direct medical costs refer to the medical cost associated with the fatal fall event. Lifetime work loss cost figures were derived from lost wages, benefits, and self-provided household services accounting for age (i.e., the lifetime work loss cost is higher for a younger individual).

Data from The Dartmouth Atlas Project was obtained as part of our predictive analysis of health care intensity (available at: https://www.dartmouthatlas.org/). The Dartmouth Atlas Project analyzes how medical resources are allocated and used throughout the United States. Medicare and Medicaid data are used to characterize national, regional, and local health care markets. We mined geographic discharge rates (medical, surgical, hip fractures), care events during the last 6 months of life (hospital admissions, intensive care unit admissions), and claims-based Medicare reimbursements (inpatient/hospital, outpatient care, skilled nursing facility, home health, hospice, physician, and total Medicare reimbursements) over the period of 2001–2018.

### Descriptive analyses

Aggregate statistics were tabulated. Sub-analyses of fatal fall events relevant to sex, race, age, and state were performed. Means and variance were calculated where appropriate. Tests of normality were performed to select the appropriate statistical algorithm for sex (i.e., Student’s T-Test for the male versus female age-adjusted death rates) and race (e.g., Wilcoxon for the race group pairwise age-adjusted death rate tests). Statistical significance was set at *p* <  0.05, and the *p*-value was adjusted to account for multiple pairwise tests.

### Predictive analytics

Multiple models were developed to predict falls rates. Linear regression analyses were performed on the longitudinal sex, age, race, and state data over 2001–2018 to produce estimates on change in annual death rates. State-based measures of health care resource distribution and utilization from the Dartmouth Atlas Project were used to predict age-adjusted falls rates. To achieve this prediction, state-based age-adjusted death rates were combined with the Dartmouth Atlas Project data and trained on six machine learning algorithms. Prior to training, the data were transformed, and missing data was imputed using k-nearest neighbor imputation. To produce an accurate prediction estimate, we employed repeated 10-fold cross-validation resampling with 10 repeats. Repeated cross-validation involves splitting the data into k-folds then repeating the cross-validation. The final model accuracy is taken as the mean from the number of repeats. Within each algorithm’s training loop, hyperparameters were optimized using an automated grid search. All statistical analyses were completed in the R programming language (R Core Team, Vienna, Austria; packages: *caret*, *psych*, *tidyverse*, *ggplot2*). This analysis was completed using publicly available aggregated data sourced from the CDC and The Dartmouth Atlas, and thus local formal institutional review board review not required.

## Results

The WISQARS database reported 465,486 total deaths due to unintentional falls with crude and age-adjusted rates of 8.42 and 7.76 per 100,000 population, respectively, spanning the period of 2001 to 2018.

### Geographic health care utilization analysis

Mean annual age-adjusted death rates from falls varied from a high of 15.3 (Wisconsin) to a low of 3.9 (Alabama) per 100,000 population over 2001–2018 (Supplemental Table 1). Estimates of annual change in death rates ranged from 0.70 (Oklahoma) to 0.03 (Georgia).

Of the six machine learning algorithms tuned & trained to predict the age-adjusted death rates based on the States’ Dartmouth Atlas data, the Elastic Net (*Enet*) algorithm was the worst performer (Supplemental Table 2; Supplemental Fig. S1). Using the trained Cubist model, the most influential variable in the prediction was hospital admissions in the last 6 months of life (Table 1).

**Table 1:** Top five most influential Dartmouth Atlas variables from the *Cubist* model prediction of age-adjusted death rates from falls over 2001–2018. Influence values scaled to 0–100

Variable Rank	Variable	Influence Value
1	Hospital Admissions, Last 6 Months of Life	82.0
2	Medicare Reimbursements for Hospice	54.0
3	Medicare Hospitalizations for Hip Fractures	45.0
4	ICU Admissions, Last 6 Months of Life	40.5
5	Medicare Reimbursements for Skilled Nursing Facilities	40.0

*ICU* intensive care unit

### Sex analysis

**Fig. 1:**
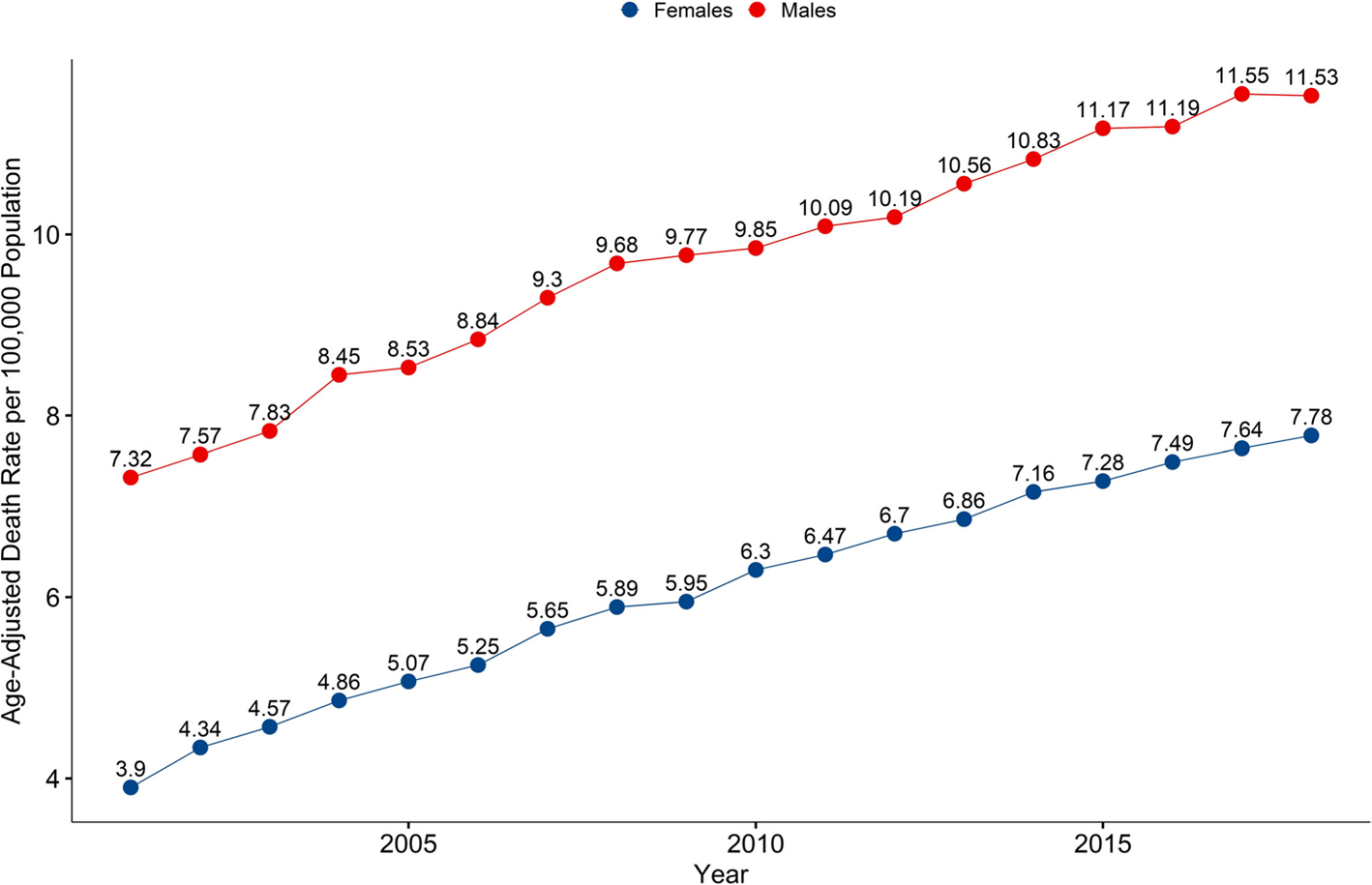
Annual United States age-adjusted falls mortality rate per 100,000 population over 2001–2018

Over the study period, the overall crude and age-adjusted death rates from falls for males were 8.72 and 9.89 per 100,000 population, respectively. For females, the overall crude and age-adjusted death rates from falls were 8.14 and 6.17 per 100,000 population, respectively. Comparing age-adjusted rates, males had a significantly higher age-adjusted death rate from falls (9.89 vs. 6.17; *p* <  0.00001). Linear regression modelling (Age − Adjusted Rate = *β* Year + *α*) on longitudinal data (Fig. 1) determined that both male and female annual age-adjusted falls mortality rates are expected to rise (Male: + 0.25 rate/year, R^2^ = 0.98; Female: + 0.22 rate/year, R^2^ = 0.99).

### Age analysis

**Fig. 2:**
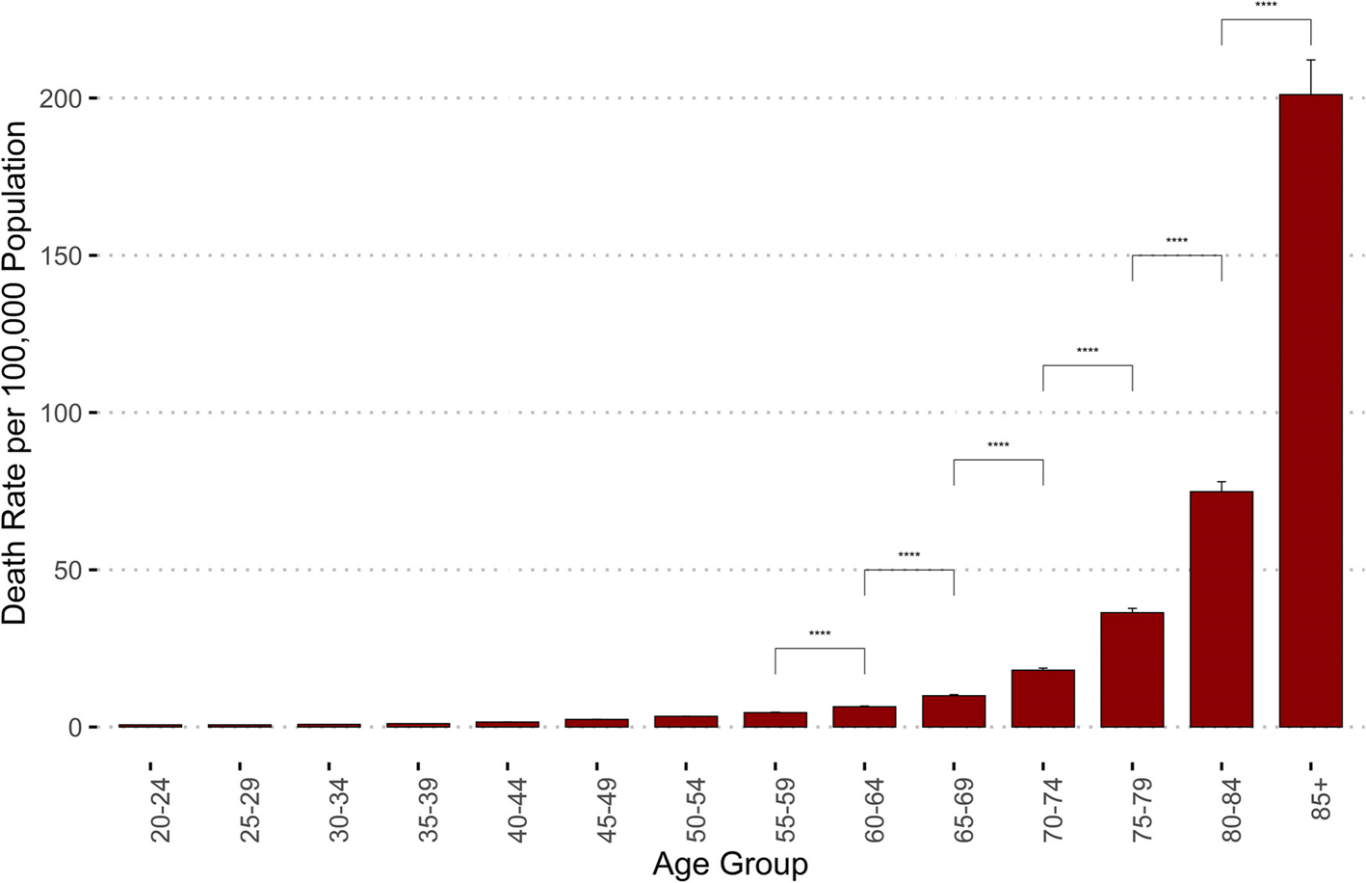
Death rates from falls in adults over 2001–2018. Error bars reflect standard error. Multiple paired comparisons between age strata were completed using Wilcoxon tests with adjusted *p*-values. ns: *p* > 0.05, *: *p* < = 0.05, **: *p* < = 0.01, ***: *p* < = 0.001, ****: *p* < = 0.0001

Adult falls mortality rates compiled from 2001 to 2018 demonstrated significant increases between age strata commensurate with increasing age (Fig. 2**).** Linear regression analysis on the longitudinal data demonstrated that age strata above 55 to 59 are predicted to have growth in annual death rates (Table 2).

**Table 2:** Adult death rates from falls per 100,000 over 2001–2018 by age strata with regression predictions of annual death rate change

Age Group	Mean Death Rate Per 100,000 (SE)	Range	Regression Estimate (β)	t value	Pr (>|t|)	R^**2**^
20–24	0.7 (0.02)	0.5–0.9	−0.02	− 0.3	0.75	0.71
25–29	0.7 (0.02)	0.6–0.9	−0.01	− 0.2	0.84	0.46
30–34	0.8 (0.02)	0.7–0.9	0.00	0.0	0.99	0.001
35–39	1.1 (0.02)	0.9–1.2	−0.01	−0.2	0.83	0.28
40–44	1.6 (0.03)	1.3–1.8	−0.02	−0.4	0.65	0.66
45–49	2.4 (0.04)	2.0–2.7	−0.02	−0.4	0.73	0.27
50–54	3.4 (0.07)	2.7–3.8	0.05	1.1	0.28	0.68
55–59	4.6 (0.2)	3.3–5.6	0.12	2.6	**0.009**	0.91
60–64	6.4 (0.2)	4.9–8.0	0.17	3.6	**0.0004**	0.97
65–69	9.9 (0.3)	7.8–12.5	0.26	5.5	**< 0.00001**	0.96
70–74	18.0 (0.6)	12.3–22.2	0.49	10.4	**< 0.00001**	0.93
75–79	36.3 (1.4)	25.1–44.7	1.09	23.0	**< 0.00001**	0.95
80–84	74.8 (3.1)	50.0–91.7	2.46	51.8	**< 0.00001**	0.97
85+	201.1 (11.0)	124.1–270.5	8.74	184.2	**< 0.00001**	0.997

*Pr* probability, *R*^2^ coefficient of determination. Bold font emphasizes *p* < 0.05

### Race analysis

Over the study period, the mean age-adjusted death rates from falls for subjects identifying as non-Hispanic and American Indian were 8.63 (std. error: 0.28), White 8.25 (std. error: 0.37), Asian/Pacific islander 4.86 (std. error: 0.13), and Black 3.88 (std. error: 0.09) per 100,000 population (Supplemental Table 3). Comparing between racial groups, annual death rates from falls were significantly different (adjusted *p* <  0.0001) except for ‘White’ versus ‘American Indian’ (adjusted *p* = 0.46) and ‘American Indian/Hispanic’ versus ‘Black/Hispanic’ (adjusted *p* = 0.49). Linear regression analysis determined that all but Black/Hispanic groups were predicted to have significant growth in annual falls mortality rates.

### Cost data

Per fatal fall event, the direct medical costs were $26,689.98 USD, and a lifetime work loss cost of $296,677.74 USD indexed to 2017 prices. Over the 2001–2018 study period, the mean annual direct medical cost was $671,419,471. (std dev. 178,449,053.) and mean annual lifetime work loss cost was $7,463,294,941. (std dev. 1,983,584,776.) (Supplemental Table 4). The total aggregate direct medical costs for 427,656 fatal fall events was $11,414,131,000.00 USD. The total lifetime work loss cost was $126,876,014,000.00.

## Discussion

In this Centers of Disease Control mortality database study covering 2001–2018, the number of unintentional fall deaths exceeded 450,000 with a combined medical and lifetime work loss cost of over $138 billion dollars. Disparities in death rates exist between sex, racial, ethnic, and age groups with older adults bearing the brunt of the highest death rates. Geographic variability in death rates also exists with geographic healthcare resource utilization intensity being predictive of age-adjusted death rates. Given the predictive power of health care intensity and persistently high death rates in older adults that continued to accelerate over the study period, the findings of this study suggest that enhanced public health initiatives to curb fatal falls in older adults should be considered.

Our findings of significant geographic variation in fall mortality reinforces the geographic context of health outcomes. Specifically, there is a nearly four-fold difference in mortality between the states with the highest (Wisconsin) and lowest (Alabama) death rates. Regional variation in health outcomes is a complex interplay of health care access, resources, and determinants of health. To our knowledge, our analysis is among the first to further explore geographic variation in mortality from unintentional falls using health care resource utilization and reimbursement data. Regional health care utilization intensity has been correlated with health outcomes ranging from efficacy in chronic disease management for Medicare patients [25], outcomes following major surgery [26], and variation in surgical care at end-of-life [27]. The predictive power of geographic intensity of health care utilization and reimbursement for falls mortality introduces the possibility of system-level levers for policy influence. Further work to investigate the relationship between health care intensity and falls mortality may uncover precise targets for systematic efforts to reduce death rates. Moreover, our analysis using the health care resource utilization and reimbursement data was not stratified by race which may also add significant contribution to the observed variation.

For adult deaths due to an unintentional fall, several demographic factors were associated with higher trends in annual death rates. Among the most striking was the relationship between death rates and age. In older adults, the aggregate longitudinal data suggested an exponential increase in death rates by increasing age, that persists after accounting for aging demographic trends. The oldest age strata, 85 years and older, was predicted to observe an accelerated increase in death rate. Our finding that death rates from falls is higher in older adults reinforces several prior analyses that have demonstrated similar trends [1–3, 21, 22, 28–30]. One possible explanation for the increasing rates of fall mortality is improved treatment of other major drivers of mortality such as stroke, heart disease, and cancer. Despite successful immediate treatment, resulting frailty and polypharmacy will subsequently increase the number of patients at risk for fall. The predictive influence of recent hospital admissions and hospice utilization seen in our study compliments this theory. Risk factors for falls in older adults are well established and include disparate factors such as gait/balance disturbance, polypharmacy, hypotension, frailty, cognitive decline, degenerative neurologic disorders, visual impairment, limitations in daily activity, environmental hazards and a history of prior falls [4–12, 31, 32]. Further research can now be targeted to identify the drivers of high yield care utilization and subsequent falls for comprehensive intervention.

To date, various public health initiatives have been developed targeting falls risk prevention in older adults in the form of clinical algorithms, prevention guidelines, and targeted mobility interventions [15–20]. In particular, the aging vestibular system in the role of falls in older adults has been a specific focus [33–41]. Despite these efforts, the longitudinal data suggests that mortality from unintentional falls is a persistent and growing public health issue. Data availability for fall prevention utilization is currently limited in existing databases, preventing a similar process of modelling that was utilized in this study. While waiting for the data to become more readily available, immediate research is warranted to analyze the quality and effectiveness of fall prevention efforts scaled to geographic adoption.

Further to the age-dependent mortality rate trends, comparison of age-adjusted death rates from falls to sex and race demographic data also demonstrated significant differences. Comparing sex, males had a significantly higher age-adjusted death rate from falls, but both sexes were predicted to have an annual increase in death rates. This finding is in support of other work from historical cohorts that demonstrated male sex is associated with higher death rates from falls [2, 42]. Comparisons between racial groups demonstrated that ‘White’ and ‘American Indian’ had the highest aggregate death rates over the study period. This result reproduces prior findings that the ‘White’ and ‘American Indian’ race groups tend to have the highest mortality rates [1, 8, 21]. Several confounders might explain sex and inter-racial differences such as access to care or reporting bias. Among different races, the circumstances of the fall event may be different in terms of the location and time of day [43–45]. In the case of sex, females are more likely to report a fall, seek care for a fall, or discuss falls prevention with their health care provider [46]. It is plausible that openly discussing falls and falls prevention may enable earlier intervention to prevent a future fatal fall. Causal mechanisms underlying these relationships are not clear and worthy of further study.

If the total combined medical and work life loss costs due to unintentional falls over the study period were compared against the gross domestic productivity of entire nations, the economic figure would equate to the world’s 58th largest economy in 2019 [47]. Other works have reproduced that costs related to falls in older adults eclipses the billions mark annually in the United States [13, 14, 45]. Beyond the meaningful benefit of reducing avoidable mortality from unintentional falls, the economic burden provides a strong financial incentive and return-on-investment argument for expansion of nationally organized falls prevention programming. In support of this concept, one study demonstrated that a multifactorial fall prevention intervention was cost effective compared to standard care for community-dwelling adults, particularly for adults aged 75 years and older [48].

This study has important limitations that need mentioning. Principally, this analysis is a descriptive study of aggregate statistics. The CDC WISQARS database does not include clinical context for the fatal fall events. Falls can occur for several reasons ranging from stumbling or a transient loss of balance to contributions from vestibular, neurologic, cardiovascular, ophthalmologic, or pharmacologic disorders. Other variables that could contribute to fall risks pertaining to race as a social construct such as housing, living arrangements, and access to resources are also not cataloged in the CDC WISQARS database. Furthermore, variation in coding practices and behaviors between states, and over time, ascribing fall as the cause of mortality could result in erroneous differences. To inform effective public health policy, further study of the top causes of unintentional falls will be necessary. Second, the conclusions from our analyses are anchored to assumptions in the quality of the administrative data. With respect to the geographic analyses of care utilization, we are unable to account for possible confounders that may influence utilization such as population composition, access to healthcare services and other potentially relevant socioeconomic factors. We assume that that deaths from unintentional falls were coded correctly with a fall being the principal mechanism of death. There is likely a degree of error in coding as determining an exact and principal cause of death can be difficult to ascertain in patients with complex medical histories and co-morbidities. Third, the incidence of fatal falls is directly associated with the quality of reporting. It is plausible that the increased efforts to report fatal falls over time is, to some degree, contributing to the increase in deaths attributable to unintentional falls [49].

## Conclusions

Geographic healthcare utilization is predictive of age-adjusted falls mortality. Over 2001 to 2018, over 450,000 people died from an unintentional fall with an economic cost exceeding $138 billion dollars. Adults aged 85+ years carried the highest death rate, and this rate is forecasted to accelerate. Further study of the relationship between geographic healthcare resource utilization and mortality from unintentional falls may inform enhanced public health policy efforts to mitigate the growing risk of death from unintentional falls in older adults.

## Supplementary Information

**Additional file 1: Supplemental Table 1**. Adult age-adjusted death rates from falls per 100,000 over 2001–2018 by State with linear regression predictions of annual death rate change. **Supplemental Table 2**. Prediction performance of six supervised machine learning models in predicting age-adjusted death rates from falls, utilizing States’ Dartmouth Atlas variables. Units of mean absolute error (MAE) and root-mean squared error (RMSE) are in age-adjusted death rate. **Supplemental Table 3.** Adult death rates from falls per 100,000 over 2001–2018 by Race & Ethnicity group strata with linear regression predictions of annual death rate change. **Supplemental Table 4.** Direct medical and lifetime work loss costs associated with falls in all ages, 2001–2018.

**Additional file 2: Supplemental Fig. S1**. Performance characteristics of the six machine learning algorithms trained on the Dartmouth Atlas and State age-adjusted death rates.

## Data Availability

Data used in this analysis is publicly available via these two repositories: ■ CDC Web-based Injury Statistics Query and Reporting System (WISQARS): https://www.cdc.gov/injury/wisqars/fatal.html ■ Dartmouth Atlas: https://data.dartmouthatlas.org/

## Abbreviations

CDC: Centers for Disease Control
Enet: Elastic Net
ICD: International Classification of Disease
NCHS: National Center for Health Statistics
STEADI: Stopping Elderly Accidents, Deaths, and Injuries
WISQARS: Web-based Injury Statistics Query and Reporting System database

## References

[1] Bergen G, Stevens MR, Burns ER (2016). Falls and Fall Injuries Among Adults Aged ≥65 Years - United States, 2014. MMWR Morb Mortal Wkly Rep.

[2] Cheng P, Wang L, Ning P (2019). Unintentional falls mortality in China, 2006-2016. J Glob Health.

[3] Hartholt KA, van Beeck EF, van der Cammen TJM (2018). Mortality from falls in Dutch adults 80 years and older, 2000-2016. JAMA.

[4] Campbell AJ, Borrie MJ, Spears GF (1989). Risk factors for falls in a community-based prospective study of people 70 years and older. J Gerontol.

[5] Dunn JE, Rudberg MA, Furner SE, Cassel CK (1992). Mortality, disability, and falls in older persons: the role of underlying disease and disability. Am J Public Health.

[6] Ganz DA, Bao Y, Shekelle PG, Rubenstein LZ (2007). Will my patient fall?. JAMA.

[7] Jia H, Lubetkin EI, DeMichele K, Stark DS, Zack MM, Thompson WW (2019). Prevalence, risk factors, and burden of disease for falls and balance or walking problems among older adults in the U.S. Prev Med.

[8] Nevitt MC, Cummings SR, Hudes ES (1991). Risk factors for injurious falls: a prospective study. J Gerontol.

[9] Nevitt MC, Cummings SR, Kidd S, Black D (1989). Risk factors for recurrent nonsyncopal falls. A prospective study. JAMA.

[10] Rubenstein LZ, Josephson KR (2006). Falls and their prevention in elderly people: what does the evidence show?. Med Clin North Am.

[11] Speechley M, Tinetti M (1991). Falls and injuries in frail and vigorous community elderly persons. J Am Geriatr Soc.

[12] Tinetti ME, Speechley M, Ginter SF (1988). Risk factors for falls among elderly persons living in the community. N Engl J Med.

[13] Florence CS, Bergen G, Atherly A, Burns E, Stevens J, Drake C (2018). Medical costs of fatal and nonfatal falls in older adults. J Am Geriatr Soc.

[14] Haddad YK, Bergen G, Florence CS (2019). Estimating the economic burden related to older adult falls by state. J Public Health Manag Pract.

[15] Panel on Prevention of Falls in Older Persons, American Geriatrics Society and British Geriatrics Society. Summary of the Updated American Geriatrics Society/British Geriatrics Society clinical practice guideline for prevention of falls in older persons. J Am Geriatr Soc. 2011;59(1):148–57. 10.1111/j.1532-5415.2010.03234.x 21226685

[16] Casey CM, Parker EM, Winkler G, Liu X, Lambert GH, Eckstrom E (2017). Lessons learned from implementing CDC’s STEADI falls prevention algorithm in primary care. Gerontologist.

[17] Houry D, Florence C, Baldwin G, Stevens J, McClure R. The CDC Injury Center’s response to the growing public health problem of falls among older adults. Am J Lifestyle Med. 2016;10(1). 10.1177/1559827615600137. 10.1177/1559827615600137 PMC4681302 26688674

[18] Gillespie LD, Robertson MC, Gillespie WJ (2012). Interventions for preventing falls in older people living in the community. Cochrane Database Syst Rev.

[19] Guirguis-Blake JM, Michael YL, Perdue LA, Coppola EL, Beil TL (2018). Interventions to prevent falls in older adults: updated evidence report and systematic review for the US preventive services task force. JAMA.

[20] Liu-Ambrose T, Davis JC, Best JR (2019). Effect of a home-based exercise program on subsequent falls among community-dwelling high-risk older adults after a fall: a randomized clinical trial. JAMA.

[21] Alamgir H, Muazzam S, Nasrullah M (2012). Unintentional falls mortality among elderly in the United States: time for action. Injury.

[22] Hartholt KA, Lee R, Burns ER, van Beeck EF (2019). Mortality from falls among US adults aged 75 years or older, 2000-2016. JAMA.

[23] Lawrence B, Bhattacharya S, Zaloshnja E, Jones P, Miller T, Corso PS (2011). Medical and Work Loss Cost Estimation Methods for the WISQARS Cost of Injury Module. Pacific Institute for Research and Evaluation (PIRE).

[24] Finkelstein EA, Corso PS, Miller TR (2006). The incidence and economic burden of injuries in the United States.

[25] Bronner KK, Wennberg JE, Fisher ES (2008). Tracking the care of patients with severe chronic illness: the Dartmouth atlas of health care 2008.

[26] Sheetz KH, Dimick JB, Ghaferi AA (2014). The association between hospital care intensity and surgical outcomes in medicare patients. JAMA Surg.

[27] Kwok AC, Semel ME, Lipsitz SR (2011). The intensity and variation of surgical care at the end of life: a retrospective cohort study. Lancet.

[28] Moudouni DKM, Phillips CD (2013). In-hospital mortality and unintentional falls among older adults in the United States. J Appl Gerontol.

[29] Mosenthal AC, Livingston DH, Elcavage J, Merritt S, Stucker S (1995). Falls: epidemiology and strategies for prevention. J Trauma.

[30] Allen CJ, Hannay WM, Murray CR (2015). Causes of death differ between elderly and adult falls. J Trauma Acute Care Surg.

[31] Hoffman GJ, Liu H, Alexander NB, Tinetti M, Braun TM, Min LC (2019). Posthospital fall injuries and 30-day readmissions in adults 65 years and older. JAMA Netw Open.

[32] Lopez D, McCaul KA, Hankey GJ (2011). Falls, injuries from falls, health related quality of life and mortality in older adults with vision and hearing impairment--is there a gender difference?. Maturitas.

[33] Agrawal Y, Carey JP, Della Santina CC, Schubert MC, Minor LB (2009). Disorders of balance and vestibular function in US adults: data from the National Health and nutrition examination survey, 2001-2004. Arch Intern Med.

[34] Bermúdez Rey MC, Clark TK, Wang W, Leeder T, Bian Y, Merfeld DM (2016). Vestibular perceptual thresholds increase above the age of 40. Front Neurol.

[35] Dieterich M, Brandt T (2019). Perception of verticality and vestibular disorders of balance and falls. Front Neurol.

[36] Ganança FF, Gazzola JM, Aratani MC, Perracini MR, Ganança MM (2006). Circumstances and consequences of falls in elderly people with vestibular disorder. Brazilian J Otorhinolaryngol.

[37] Herdman SJ, Blatt P, Schubert MC, Tusa RJ (2000). Falls in patients with vestibular deficits. Am J Otol.

[38] Murray KJ, Hill K, Phillips B, Waterston J (2005). A pilot study of falls risk and vestibular dysfunction in older fallers presenting to hospital emergency departments. Disabil Rehabil.

[39] Pothula VB, Chew F, Lesser THJ, Sharma AK (2004). Falls and vestibular impairment. Clin Otolaryngol Allied Sci.

[40] Sloane PD, Baloh RW, Honrubia V (1989). The vestibular system in the elderly: clinical implications. Am J Otolaryngol.

[41] Zalewski CK (2015). Aging of the human vestibular system. Semin Hear.

[42] Alamgir H, Muazzam S, Nasrullah M (2012). [copy] unintentional falls mortality among elderly in the United States: time for action. Injury.

[43] Kwan MM-S, Close JCT, Wong AKW, Lord SR (2011). Falls incidence, risk factors, and consequences in Chinese older people: a systematic review. J Am Geriatr Soc.

[44] Han BH, Ferris R, Blaum C (2014). Exploring ethnic and racial differences in falls among older adults. J Community Health.

[45] Faulkner KA, Cauley JA, Zmuda JM (2005). Ethnic differences in the frequency and circumstances of falling in older community-dwelling women. J Am Geriatr Soc.

[46] Stevens JA, Ballesteros MF, Mack KA, Rudd RA, DeCaro E, Adler G (2012). Gender differences in seeking care for falls in the aged Medicare population. Am J Prev Med.

[47] World Bank (2020). World Development Indicators.

[48] Isaranuwatchai W, Perdrizet J, Markle-Reid M, Hoch JS (2017). Cost-effectiveness analysis of a multifactorial fall prevention intervention in older home care clients at risk for falling. BMC Geriatr.

[49] Kharrazi RJ, Nash D, Mielenz TJ (2015). Increasing trend of fatal falls in older adults in the United States, 1992 to 2005: coding practice or reporting quality?. J Am Geriatr Soc.

