# Supplementary material for: The predictive power of geographic health care utilization for unintentional fatal fall rates
Source: BMC Public Health. 2022 Feb 16;22:328. doi: 10.1186/s12889-022-12731-x (PMC8848674; doi:10.1186/s12889-022-12731-x)
Supplement: Supplementary file 1 — Additional file 1: Supplemental Table 1. Adult age-adjusted death rates from falls per 100,000 over 2001–2018 by State with linear regression predictions of annual death rate change. Supplemental Table 2. Prediction performance of six supervised machine learning models in predicting age-adjusted death rates from falls, utilizing States’ Dartmouth Atlas variables. Units of mean absolute error (MAE) and root-mean squared error (RMSE) are in age-adjusted death rate. Supplemental Table 3. Adult death rates from falls per 100,000 over 2001–2018 by Race & Ethnicity group strata with linear regression predictions of annual death rate change. Supplemental Table 4. Direct medical and lifetime work loss costs associated with falls in all ages, 2001–2018. [file 12889_2022_12731_MOESM1_ESM.docx]

**SUPPLEMENTAL TABLES**

**Supplemental Table 1**. Adult age-adjusted death rates from falls per 100,000 over 2001-2018 by State with linear regression predictions of annual death rate change.

| **State** | **Mean Death Rate Per 100,000 (SE)** | **Range** | **Regression Estimate (β)** | **t value** | **Pr(>\|t\|)** | **R^2^** |
| --- | --- | --- | --- | --- | --- | --- |
| Wisconsin | 15.3 (0.7) | 11.3-21.5 | 0.54 | 12.4 | 0.0001 | 0.90 |
| Vermont | 15.1 (0.9) | 4.1-20. | 0.58 | 13.3 | < 0.0001 | 0.63 |
| New Mexico | 14.5 (0.5) | 10-17.8 | 0.12 | 2.8 | 0.005 | 0.12 |
| Minnesota | 13.0 (0.6) | 9.4-17.2 | 0.50 | 11.5 | < 0.0001 | 0.97 |
| Colorado | 12.9 (0.7) | 8-16.8 | 0.56 | 12.9 | < 0.0001 | 0.95 |
| South Dakota | 12.8 (0.6) | 7.8-17.5 | 0.38 | 8.8 | < 0.0001 | 0.61 |
| Arizona | 11.5 (0.3) | 9 – 13.3 | 0.18 | 4.2 | < 0.0001 | 0.58 |
| Oregon | 11.3 (0.6) | 5.4 – 14.9 | 0.48 | 11.0 | < 0.0001 | 0.90 |
| Washington | 10.9 (0.4) | 8.4 -13.4 | 0.27 | 6.2 | < 0.0001 | 0.89 |
| Montana | 10.9 (0.4) | 6.9 -13.2 | 0.20 | 4.7 | < 0.0001 | 0.45 |
| Rhode Island | 10.7 (0.5) | 5.9 -15.1 | 0.38 | 8.7 | < 0.0001 | 0.75 |
| Idaho | 10.5 (0.4) | 7.6 -13.9 | 0.26 | 6.0 | < 0.0001 | 0.59 |
| New Hampshire | 10.5 (0.8) | 5.0 -15.8 | 0.62 | 14.2 | < 0.0001 | 0.96 |
| Iowa | 10.3 (0.5) | 6.2 -13.4 | 0.40 | 9.1 | < 0.0001 | 0.91 |
| Oklahoma | 9.7 (0.9) | 4.6 -15.8 | 0.70 | 16.1 | < 0.0001 | 0.96 |
| Wyoming | 9.6 (0.7) | 4.6 – 15.6 | 0.46 | 10.4 | < 0.0001 | 0.73 |
| Kansas | 9.2 (0.5) | 6.0 -12.9 | 0.42 | 9.5 | < 0.0001 | 0.91 |
| Missouri | 9.1 (0.2) | 7.0 – 10.4 | 0.14 | 3.2 | 0.002 | 0.53 |
| West Virginia | 9.1 (0.7) | 5.1 -14.1 | 0.54 | 12.4 | < 0.0001 | 0.93 |
| Nebraska | 8.6 (0.2) | 6.7 – 9.6 | 0.12 | 2.6 | 0.008 | 0.41 |
| North Dakota | 8.6 (0.3) | 6.4 -11.3 | 0.06 | 1.4 | 0.15 | 0.06 |
| North Carolina | 8.5 (0.4) | 5.6 – 11.1 | 0.33 | 7.5 | < 0.0001 | 0.95 |
| District of Columbia | 8.1 (0.3) | 5.8 – 10. | 0.13 | 3.0 | 0.003 | 0.31 |
| Tennessee | 8.0 (0.3) | 5.7 – 9.8 | 0.25 | 5.7 | < 0.0001 | 0.91 |
| Utah | 8.0 (0.4) | 5.2 – 10.4 | 0.25 | 5.8 | < 0.0001 | 0.69 |
| Florida | 8.0 (0.4) | 5.1 – 9.9 | 0.28 | 6.3 | < 0.0001 | 0.95 |
| Ohio | 7.9 (0.4) | 5.0 – 10.7 | 0.31 | 7.1 | < 0.0001 | 0.93 |
| Mississippi | 7.8 (0.2) | 6.1 – 9.5 | 0.14 | 3.2 | 0.002 | 0.53 |
| Georgia | 7.8 (0.1) | 6.7 – 8.9 | 0.03 | 0.6 | 0.56 | 0.05 |
| Maryland | 7.7 (0.4) | 4.3 – 10.7 | 0.34 | 7.7 | < 0.0001 | 0.95 |
| Maine | 7.7 (0.9) | 3.7 – 19.1 | 0.56 | 12.9 | < 0.0001 | 0.65 |
| Pennsylvania | 7.7 (0.4) | 4.7 – 9.8 | 0.30 | 6.8 | < 0.0001 | 0.93 |
| Virginia | 7.2 (0.4) | 4.8 – 10 | 0.32 | 7.3 | < 0.0001 | 0.93 |
| Michigan | 7.2 (0.3) | 5.2 – 9.5 | 0.23 | 5.2 | < 0.0001 | 0.91 |
| Nevada | 7.1 (0.4) | 4.2 – 10 | 0.25 | 5.7 | < 0.0001 | 0.77 |
| Texas | 7.0 (0.3) | 4.6 – 8.0 | 0.17 | 4.0 | 0.0001 | 0.74 |
| Hawaii | 7.0 (0.2) | 4.9 – 8.5 | 0.12 | 2.8 | 0.005 | 0.40 |
| Connecticut | 6.9 (0.4) | 4.3 -8.9 | 0.25 | 5.8 | < 0.0001 | 0.80 |
| Alaska | 6.4 (0.7) | 2.4 – 14.2 | 0.44 | 10.1 | < 0.0001 | 0.58 |
| Arkansas | 6.3 (0.2) | 4.6 – 8.3 | 0.15 | 3.4 | 0.0006 | 0.67 |
| South Carolina | 6.3 (0.4) | 4.4 – 9.8 | 0.28 | 6.5 | < 0.0001 | 0.90 |
| Massachusetts | 6.3 (0.5) | 3.1 – 9.9 | 0.38 | 8.8 | < 0.0001 | 0.95 |
| Illinois | 6.0 (0.3) | 4.3 – 7.7 | 0.21 | 4.8 | < 0.0001 | 0.96 |
| Kentucky | 5.9 (0.2) | 4.4 – 7.2 | 0.16 | 3.7 | 0.0002 | 0.81 |
| Delaware | 5.7 (0.3) | 3.0 – 8.0 | 0.11 | 2.6 | 0.01 | 0.29 |
| New York | 5.6 (0.1) | 4.7 – 6.6 | 0.11 | 2.6 | 0.009 | 0.91 |
| California | 5.5 (0.1) | 4.0 – 6.1 | 0.09 | 2.1 | 0.04 | 0.79 |
| Indiana | 5.4 (0.2) | 4.1 – 7.1 | 0.16 | 3.7 | 0.0003 | 0.91 |
| Louisiana | 5.0 (0.3 | 3.2 – 6.9 | 0.20 | 4.6 | < 0.0001 | 0.82 |
| New Jersey | 4.1 (0.1) | 3.0 – 4.8 | 0.10 | 2.3 | 0.02 | 0.79 |
| Alabama | 3.9 (0.1) | 2.9 – 4.7 | 0.07 | 1.6 | 0.12 | 0.65 |

Pr: probability; R^2^: coefficient of determination; SE: standard error

**Supplemental Table 2**. Prediction performance of six supervised machine learning models in predicting age-adjusted death rates from falls, utilizing States’ Dartmouth Atlas variables. Units of mean absolute error (MAE) and root-mean squared error (RMSE) are in age-adjusted death rate.

|  | **Mean (Interquartile Range)** | | | |
| --- | --- | --- | --- | --- |
| **Machine Learning Model** | **MAE** | **RMSE** | **R^2^** |  |
| Cubist | 0.92 (0.84-0.99) | 1.33 (1.20-1.45) | 0.84 (0.81-0.87) |  |
| Radial Vector Machines | 1.22 (1.15-1.32) | 1.66 (1.53-1.80) | 0.75 (0.72-0.80) |  |
| Random Forest | 1.16 (1.09-1.24) | 1.58 (1.47-1.67) | 0.78 (0.75-0.82) |  |
| Multivariate Adaptive Regression Splines | 1.65 (1.52-1.75) | 2.13 (1.96-2.26) | 0.59 (0.55-0.64) |  |
| Elastic Net | 1.90 (1.78-2.04) | 2.49 (2.34-1.65) | 0.44 (0.39-0.50) |  |
| Gradient Boosting Machine | 1.13 (1.02-1.25) | 1.56 (1.43-1.67) | 0.78 (0.73-0.82) |  |

MAE: mean absolute error; RMSE: root-mean squared error; R^2^: coefficient of determination.

**Supplemental Table 3.** Adult death rates from falls per 100,000 over 2001-2018 by Race & Ethnicity group strata with linear regression predictions of annual death rate change.

| **Racial Group** | **Mean Death Rate Per 100,000 (SE)** | **Range** | **Regression Estimate (β)** | **t value** | **Pr(>\|t\|)** | **R^2^** |
| --- | --- | --- | --- | --- | --- | --- |
| American Indian | 8.63 (0.28) | 6.4-10.4 | 0.18 | 8.7 | **< 0.0001** | 0.67 |
| American Indian (H) | 1.09 (0.14) | 0.2-2.4 | 0.07 | 3.5 | **0.0006** | 0.46 |
| Asian/Pacific Islander (H) | 2.68 (0.23) | 0.9-4.4 | 0.10 | 4.5 | **< 0.0001** | 0.88 |
| Asian/Pacific Islander | 4.86 (0.13) | 3.8-5.8 | 0.10 | 4.7 | **< 0.0001** | 0.30 |
| Black | 3.88 (0.09) | 3.2-4.6 | 0.07 | 3.2 | **0.002** | 0.83 |
| Black (H) | 1.16 (0.10) | 0.6-2.1 | 0.03 | 1.2 | 0.22 | 0.11 |
| White | 8.25 (0.37) | 5.6-10.5 | 0.29 | 13.9 | **< 0.0001** | 0.99 |
| White (H) | 5.70 (0.14 | 4.3-6.3 | 0.10 | 4.7 | **< 0.0001** | 0.78 |

(H): Hispanic; Pr: probability; R^2^: coefficient of determination; SE: standard error. Bold font emphasizes p < 0.05.

**Supplemental Table 4.** Direct medical and lifetime work loss costs associated with falls in all ages, 2001-2017.

|  |  | **Cost ($ in USD; 2017 Prices)** | | |
| --- | --- | --- | --- | --- |
| **Year** | **Fall Deaths** | **Direct Medical^*^** | **Lifetime Work Loss^#^** | **Combined** |
| 2001 | 15,015 | $ 400,750,000.00 | $ 4,454,616,000.00 | $ 4,855,366,000.00 |
| 2002 | 16,238 | $ 433,392,000.00 | $ 4,817,453,000.00 | $ 5,250,845,000.00 |
| 2003 | 17,214 | $ 459,441,000.00 | $ 5,107,011,000.00 | $ 5,566,452,000.00 |
| 2004 | 18,792 | $ 501,558,000.00 | $ 5,575,168,000.00 | $ 6,076,726,000.00 |
| 2005 | 19,622 | $ 523,711,000.00 | $ 5,821,411,000.00 | $ 6,345,121,000.00 |
| 2006 | 20,802 | $ 555,205,000.00 | $ 6,171,490,000.00 | $ 6,726,695,000.00 |
| 2007 | 22,618 | $ 603,674,000.00 | $ 6,710,257,000.00 | $ 7,313,931,000.00 |
| 2008 | 23,994 | $ 640,399,000.00 | $ 7,118,486,000.00 | $ 7,758,885,000.00 |
| 2009 | 24,761 | $ 660,871,000.00 | $ 7,346,037,000.00 | $ 8,006,908,000.00 |
| 2010 | 25,980 | $ 693,406,000.00 | $ 7,707,688,000.00 | $ 8,401,093,000.00 |
| 2011 | 27,453 | $ 732,720,000.00 | $ 8,144,694,000.00 | $ 8,877,414,000.00 |
| 2012 | 28,728 | $ 766,750,000.00 | $ 8,522,958,000.00 | $ 9,289,708,000.00 |
| 2013 | 30,188 | $ 805,717,000.00 | $ 8,956,107,000.00 | $ 9,761,825,000.00 |
| 2014 | 31,937 | $ 852,398,000.00 | $ 9,474,997,000.00 | $ 10,327,395,000.00 |
| 2015 | 33,355 | $ 890,244,000.00 | $ 9,895,686,000.00 | $ 10,785,930,000.00 |
| 2016 | 34,651 | $ 924,835,000.00 | $ 10,280,180,000.00 | $ 11,205,015,000.00 |
| 2017 | 36,308 | $ 969,060,000.00 | $ 10,771,775,000.00 | $ 11,740,835,000.00 |
| Totals: | 427,656 | $ 11,414,131,000.00 | $ 126,876,014,000.00 | $ 138,290,144,000.00 |

* Direct medical costs $26,689.98 USD per fatal fall event.
# Lifetime work loss cost of $296,677.74 USD per fatal fall event.
