# Supplementary material for: The predictive power of geographic health care utilization for unintentional fatal fall rates
Source: BMC Public Health. 2022 Feb 16;22:328. doi: 10.1186/s12889-022-12731-x (PMC8848674; doi:10.1186/s12889-022-12731-x)
Supplement: Supplementary file 2 — Additional file 2: Supplemental Fig. S1. Performance characteristics of the six machine learning algorithms trained on the Dartmouth Atlas and State age-adjusted death rates. [file 12889_2022_12731_MOESM2_ESM.docx]

**Supplemental Figure S1**. Performance characteristics of the six machine learning algorithms trained on the Dartmouth Atlas and State age-adjusted death rates.


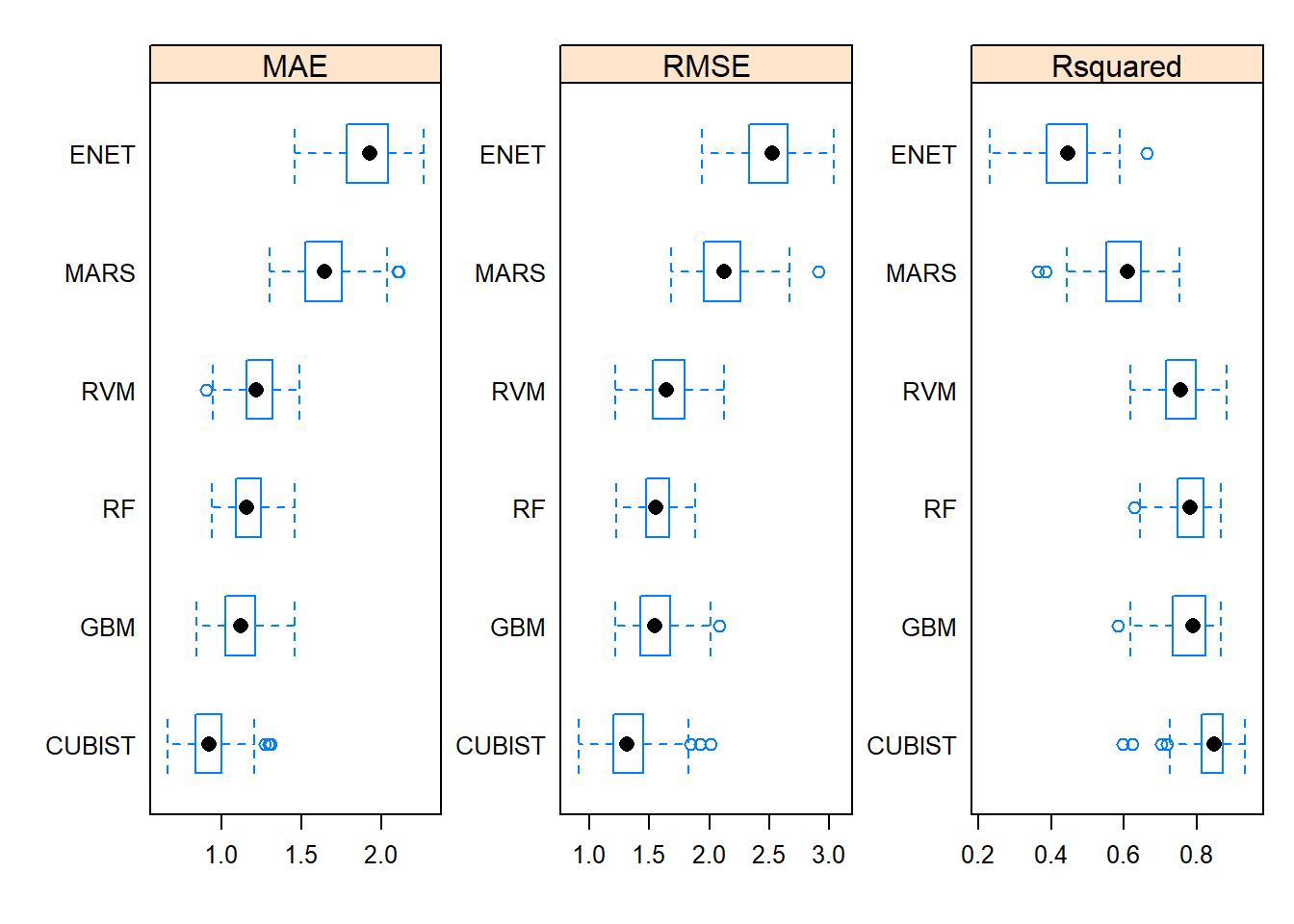


MAE: Mean Absolute Error; RMSE: Root Mean Squared Error
